# ‘ALL ABOUT MY IDEAL MENTAL HEALTH SERVICE’: Users, family members and experts by experience discussing a co‐designed service

**DOI:** 10.1111/hex.13999

**Published:** 2024-03-04

**Authors:** Michele Rocelli, Ludovica Aquili, Paolo Giovanazzi, Andrea Puecher, Marco Maria Goglio, Elena Faccio

**Affiliations:** ^1^ Department of Philosophy, Sociology, Education and Applied Psychology University of Padua; ^2^ Il Cerchio Fareassieme Trento Italy; ^3^ Director of Psychiatry Unit Trentino Italy

**Keywords:** co‐designed service, experts by experience, ideal mental health service, person‐centred care, quality service

## Abstract

**Introduction:**

Many studies have investigated patients' understandings of how to optimise mental health services. However, only a few studies in the Italian context have involved experts by experience (EbEs), who can be ex‐users, family members of ex‐users or current service collaborators. Their role is crucial in implementing collaborative service quality assessment projects.

**Method:**

The study investigated the experience of 35 EbEs,  users, and family members who carried out a 9‐month fortnightly project aimed at imagining an ‘ideal service’. The facilitators of the discussion groups (two EbEs) were interviewed; written reports of each meeting were produced with relevant comments, notes and specific suggestions; and content analysis was applied.

**Results:**

The most important result concerns the effectiveness of the project management method and group leadership carried out by the two EbEs. This approach allowed for complete autonomy of the work, without professional gaze or power imbalance. Also, the ideas and specific contents focused on by the two groups offer strategies to facilitate users' entry and reception in health care centres, to reduce the stigma of mental illness, to improve the centres' physical environment, to improve organisational aspects, to keep family members actively involved and to network mental health services with other territorial services.

**Conclusions:**

EbEs have proven to be key figures in ensuring equity of role in the service co‐design process. This also concerns a context, the Italian one, where their role has not yet been recognised and legalised. Their contribution and ideas to improve services could be fundamental not only in mental health centres, but also in other health facilities, and could concern the entire service delivery process rather than being limited to quality assurance, according to a virtuous circle based on active participation and transformation of the role of users.

**Patient or Public Contribution:**

This work resulted from close collaboration between the two EbEs who conducted the groups, users and family members, the university, and the psychiatrist in charge of the service. All of them contributed to the research. The EbEs, researchers and psychiatrist participated in the interpretation of the data and are the co‐authors of this paper.

## 1 INTRODUCTION

The World Health Organisation (WHO) European Mental Health Action Plan^1^ advocates for ‘more cooperation between mental health service users and their relatives in the reorganisation, delivery, evaluation, and monitoring of services, so that care and treatment become more responsive to their needs’. This will allow the consideration of users and their relatives as a vital part of the quality assurance process of service delivery and the formulation of health policies. This article reports on the partial outcomes of a collaborative process between the users and operators of a mental health centre in Northern Italy aimed at designing an ideal service and suggesting insights regarding the co‐designing of services.

### 1.1 The ideal mental health service

Many studies have explored users' expectations of an ideal mental health service, and many stimulating ideas have been generated. Some suggestions are aimed at different types of users, while others reflect the stage of life, interests, and needs of a specific age group. Regarding adolescents, for example, it is important that healthcare services be sensitive to their cultural backgrounds and respond appropriately in meeting their individual mental healthcare needs.^2^ To serve the different groups of adolescents seeking help most effectively, information about the services must be simple, provided in multiple languages and available online in both audible and illustrative versions. Services for adolescents must also be youth‐friendly,that is, easy to access, inexpensive and handy, with short waiting lists and flexible hours. The most effective services for adolescents are run by multiskilled health staff who maintain constant contact with them and provide appropriate support.

Other service qualities, such as the relationship between practitioners and users, may be best estimated by users of different ages. Particularly, studies that have investigated the most desirable qualities in relationships with practitioners report that a supportive and empathetic relationship bolstered by effective communication is the ideal clinical relationship with all users.^3^, ^4^, ^5^ Among the suggested tips for how to improve services, users have identified and emphasised the urgent need to be involved in the final decisions regarding their recommended treatments.^6^

Users are aware of the power dynamics in the delivery of mental health services, and they wish to assume a greater role in this process. Among the most desired aspects they identified was being part of a service that provides care in a personalised way, without stigmatisation, and that includes planned collaboration with users.^7^ According to patients, redesigning services that are focused on personalised care rather than control is a high priority, one that includes enhancing health professionals' empathetic qualities.^8^ Therefore, user involvement in co‐designing mental healthcare projects is an important characteristic of an ideal service.

### 1.2 Involving users in the quality assurance process

The literature points out the challenges associated with co‐designing projects. The first challenge involves motivational issues concerning user involvement. For example, what might persuade current or former users to take part in the exhausting and sometimes problematic process of reviewing a service? According to Repper and Breeze,^9^ this involves their sense that they are helping to improve the services and ‘giving something in return’ from their recovery path experience. Some users have described their participation as ‘cathartic’, since it provides psychological relief through the open expression of strong emotions, allowing them to learn new knowledge and empowering them in the acquisition of self‐confidence and self‐esteem. However, the users have also identified several important critical issues. First, despite the guidelines about involving service users and carers in mental health education and training, there are not enough supportive reward systems for users.^10^ Second, some users have reported that privacy issues, embarrassment and professional jargon are the main barriers to their engagement. These difficulties highlight the problem of a power imbalance in favour of professionals in the mental health field.^11^

Co‐production has been presented as a means to ameliorate the power inequalities embedded in healthcare hierarchies, in particular, those between psychiatric managers and service users; however, the manner in which it is realised determines whether these inequalities are replicated or transformed.^12^ User involvement alone cannot ensure that services will be more democratic. The chance to complain or express criticism and disagreement about the work of professionals cannot be entirely free if the practitioner has the power to express himself or herself based on the mental health condition of the user. Cowden and Singhpoint^13^ ^,p. 5^ pointed out that the value of user involvement in service design is often taken for granted, without considering ‘what it actually means to involve the user’. In addition, the concepts and methodologies used in the mental health field can cause misunderstandings among stakeholders and undermine the effectiveness of collaborative initiatives. For example, health professionals often engage users only in formal or superficial activities, with limited, if any, constructive impact.

According to Oliver et al.,^14^ these less than satisfactory results can occur, especially when users are not informed about the reasons for their involvement and the intended effects of the projects. Therefore, co‐design work requires that participants, particularly service professionals and researchers, maintain a reflective gaze on the types and ways of ‘collaboration’ and ‘involvement’ and avoid ineffective initiatives based on false collaborations. To maintain a reflective gaze means to constantly inquire about how to make service users a real part of the process of evaluating service quality, how to facilitate their free expression without compromising the relationships and services they need, and how to manage the disparities in power between users and professionals.

The literature shows that in attempts to involve users and families in the development of healthcare services, the user experience is at the core. In the 1960s and 1970s, the main approach was to involve users through traditional research methods, such as surveys and interviews.^9^, ^15^ However, although these tools offer users the opportunity to express their perceptions and needs, their ability to generate genuine and fruitful involvement has been questioned because even the wording and structure of the questions—very important elements for research—are the product of the point of view of those who conceived them, that is, professionals, and can reproduce role asymmetry. Hence, these methods do not lead to substantial user involvement unless the service users participate actively from the very beginning of the research design.^16^ Furthermore, this approach does not ensure the epistemic leap^17^ that the projects aim to achieve, that is, the shifting of the view of the user from a patient to an expert connoisseur of the services they use.^18^

In recent years, this view of user involvement has shifted decisively towards models in which the user is seen as an active collaborator. This change is clearly reflected in the UK's public participation charity, called INVOLVE's, and in its definition of ‘involvement’ which frames health research and service development as activities developed ‘with’ or ‘by’ service users, in contrast to other approaches that objectify users. Thus, INVOLVE emphasises the need for an evolution in the methodologies and concepts used in research and service development practices to ensure that they reflect collaboration and co‐production with user communities.^19^

From our perspective, to optimise users' involvement, it is vital that they liaise with the services they receive in such a way that they feel a sense of belonging in the process. In this scenario, they would not fear repercussions due to their expression of negative comments or complaints directed towards health professionals.

This research presents an experience built and designed by a group of experts by experience (EbEs). The terms ‘Expert by Experience’ (EbE) and ‘Peer Specialist’ refer to someone who is experienced in dealing with mental distress, either directly or in one's family. Such individuals have embarked on their recovery paths and completed specific training that allowed them to build knowledge based on their experience and develop self‐esteem and awareness in the process.^20^, ^21^, ^22^ The unique characteristics of EbEs facilitate the development of users' recovery pathways, which are focused on ‘relational recovery’.^23^ The integration of these professionals in the collaboration process and the development of a coherent and structured methodology for active involvement are crucial pillars on which to build health services that are truly user‐oriented, by understanding, valuing and integrating the knowledge EbEs have gained along their care and recovery pathways.

#### 1.2.1 The EbE role and its potential function in mental health services

Many studies worldwide have reported consistent evidence of improvement in the recovery of patients with psychiatric disorders after introducing EbEs in the process.^24^, ^25^, ^26^ Services addressing addiction disorders were the first to include former patients in assertive care teams and training programmes for job placement, with good and excellent results.^27^, ^28^, ^29^, ^30^, ^31^ In psychiatry, beneficial effects of the participation of EbEs have been reported at different levels:

1. EbEs offer unique opportunities for recognising and reframing an illness within a patient's life history, mainly because the process involves a comparison with an ‘other like me’.^21^, ^32^ It has been shown that receiving social support from individuals who have faced and overcome similar challenges in life fosters a sense of efficacy in people and positively influences various aspects of their experience.^33^
2. EbEs act as intermediaries in the provision of professional support services, balancing the asymmetrical relationships between patients and psychiatrists or caregivers and emphasising the relational aspect.^20^
3. This relationship offers greater opportunities for users to share their experiences, which leads to their better understanding, affiliation and empowerment, which is rarely achieved in traditional clinical relationships.^34^, ^35^

EbEs do not aim to replace traditional practitioners; instead, they strive to provide ‘equal’ relationships that humanise care processes. In some Anglo‐Saxon contexts, EbEs maintain a link with mental health services through the support of mentor operators.^36^ EbEs also play an active role in the decision‐making processes related to service improvement, as they participate in quality assurance comparison groups.

### 1.3 The study's aims

This research had two aims. The first was to explore the experiences of a group of 35 people, including EbEs, users and family members, who carried out a 9‐month fortnightly project with the aim of imagining an ‘ideal service’. The second aim was to reflect on and identify the key elements in the continued involvement of users and family members in the quality assurance process of mental health services, based on their experience, that is, as EbEs.

### 1.4 Design

A qualitative research approach was adopted, following a grounded theory perspective, as we were interested in understanding people's thoughts, attitudes, and personal experiences with respect to the service use, in its pros and cons. The motivation for preferring a qualitative methodology stem from the fact that we wanted to investigate the phenomenon in its natural context, giving due prominence to people's meanings, experiences and points of view. In addition, the material collected was extremely varied (narratives derived from interviews, transcriptions of focus group, facilitators' notes) and required a very flexible and adaptable method of analysis, able to make sense of, but also to give order to its complexity.

## 2 MATERIALS AND METHODS

### 2.1 Context

In the Italian context, attention to the integration of EbEs in mental health services is almost nonexistent, far behind European standards. Only a few mental health service providers have begun to respond to the WHO directives, taking steps to introduce these individuals (Table 1).

**Table 1:** Notes on the Italian context with respect to the role of the EbE.

Italy is still at the beginning of the EbE adventure. The National Charter of Peer Support Experts was a result of the First National Conference of Users and Families, which was held in 2021. It was attended by 43 local representatives from 11 Italian regions who had experience in mental health services and were active in providing peer support. In 2023, the first national training course for EbEs was launched by the Ministry of Health, and the National Network of Experienced Peer Support Users and Family Members was established. However, the process of recognition and legitimisation of the EbE role is only in its initial stages.

Abbreviation: EbE, expert by experience.

An example is the EbE network that is active in the mental health services of the Azienda Sanitaria di Trento, located in Northern Italy.^37^ Its experience in service design is presented as follows.

To fully understand the context and objectives of this research, it is helpful to delineate two distinct phases. Phase 1 describes a project that involved users, family members and EbEs of the Trento service, commissioned by the psychiatrist in charge of the service, to conduct two focus groups on the theme ‘the ideal service’. The university was also involved in Phase 2, working with the two EbEs who led the groups, to analyse the collected data, discuss it and collaborate on the drafting of this paper.

#### 2.1.1 Phase 1: ‘The ideal service’: The service design experience of users, families and EBEs

The mental health services department of the city of Trento has promoted the establishment of a group called ‘Il Cerchio Lab’ (composed of EbEs, users and family members) with the aim of implementing new practices for the active participation of users. Among the various projects this group has undertaken, one in particular, is oriented towards gathering the opinions of users, family members and EbEs on how to imagine an ideal mental health service.

### 2.2 Participants and recruitment

The participants (9 users, 10 family members and 16 EbEs) were recruited by the two EbE members of Il Cerchio Lab (1 user EbE and 1 family EbE, who are the co‐authors of this article), with the intention of involving participants with heterogeneous characteristics. The inclusion criteria shared between the EbEs and the psychiatrist member of Il Cerchio Lab were as follows: users have already been received by the service, family members have not had contact with the EbEs, ex‐users with a long relationship with the service, users or ex‐users with different mental pathologies, different ages, men and women and different levels of education. The two EbEs were assisted by the service secretariat in recruiting the participants, organising a presentation of the project, and collecting the actual sign‐ups. Afterwards, they led the discussion groups.

It should be emphasised that the only ‘service workers’ who participated in the group were the EbEs, in agreement with the mental health service. This was to safeguard their full freedom of speech, which would have been compromised if other power roles within the service (psychiatrist, nurses or educators) had also participated in the group. All group participants (conductors, EbEs, users and family members) received hourly compensation—funded by the mental health services of Trento—for participating in the discussion groups. The following table summarises the participants' demographic data.

The working and recruiting methodology adopted consisted of the psychiatric approach of ‘FAREASSIEME’^38^ a perspective that promotes the involvement of users, clinicians, operators and family members in the co‐creation of mental health service projects. Consistent with Franco Basaglia's approach,^39^, ^40^ the participants adopted participatory discussion as a method of fulfilling the mandate. The discussions had not only research but also socio‐political value due to both the objectives and the involvement of the project participants. The 35 participants (see Table 2) shared the desire to create mixed groups of users and family members to compare different points of view. Two groups were created for the discussions, both of which were facilitated by the EbEs. The meetings took place every 15 days, for a total of 15 + 15 meetings, from October to May 2021/2022 at the Il Cerchio Lab premises. The duration of each meeting was approximately 2 h.

**Table 2:** Summary of the demographic features of the study participants.

Demographic data	Demographic features	Participants (35)
Gender	Men	17
	Women	18
	Other	0
Age	18–40	7
	41–60	11
	>61	17
Origin	Trento	35
	Other	
Occupation	Employees	10
	Disabled/invalids	5
	Retired	16
	Students	4
	ESP	16
	Users	9
	Ex‐user	0
	Family members	10

The aim of the first meeting was to identify themes for discussion across the two groups on the question: What is my ideal service? Three areas of exploration emerged (see Table 3):

1. Perceptions and attitudes towards the service before and after access.
2. Analysis of critical issues and resources regarding the service.
3. Conceptualisation of an ideal mental health service.

**Table 3:** Objectives and related questions asked during the focus groups.

Objectives	Some of the questions asked
Perceptions and attitudes towards the service before and after access	WHAT DID I KNOW BEFORE?
	What did you know about mental illness and mental health services before your personal involvement?
	What experiences did you have regarding mental health before your participation?
	What did it bring to your mind? What facts or memories were elicited by your experiences?
	WHAT CHANGED AFTERWARDS?
	How did your perceptions and attitudes about mental illness and mental health services change after you experienced them personally or observed them in your family?
	What did it mean to experience these from the inside, in your own skin?
	What did this direct experience cause you to discover that you did not know or did not think about before?
Analysis of critical issues and resources regarding the service	BENEFITS: Can you describe any positive experiences you had in your interaction with the mental health service? Did you recognise any good practices?
	DEFECTS: Can you identify any shortcomings, demerits, criticisms or problems you faced dealing with the service after your inclusion?
Conceptualisation of an ideal mental health service	In light of your own experience of mental illness, what would your ideal mental health service look like if you could realise it? How would you describe it?
	How would it be different from the current one?
	If you had no financial/human resource constraints, what would your vision of mental health services look like?

Based on the answers to above questions, the suggestions and initiatives regarding the improvement of the quality of service were focused on feasibility, originality, efficiency and effectiveness.

The two groups, A and B, worked in parallel. The ideas that emerged in one group were also proposed and discussed in the second group, initiated by the presenters, who were the same two EbEs (P. G. and A. P.). Phase 1 resulted in a book published in 2022 entitled *Psychiatry as a Protagonist*,^37^ consisting of the ideas of the 30 participants in narrative and discursive form on the subject of an ideal service. The book presents the participants' extracts and testimonies in an extensive and informative manner, with comments and references by the authors.

#### 2.2.1 Phase 2: Co‐produced research

In this second phase, the composition of the research team, the collaborative methodology implemented for the data analysis, the selection of the most important findings, the discussion and the design of this article are presented.

### 2.3 Research team

The research team consisted of three researchers from the University of Padua, two EbEs (the facilitators of the two groups in the ‘my ideal service’ project) and the psychiatrist in charge of the service. The three researchers were involved in collecting, systematising and analysing the extensive material that emerged from the meetings. The collaboration was focused on answering the research question, exploring the relevant scientific literature and sharing the results of the data analysis. The sharing of the purpose, material, analysis and writing of the paper followed the guidelines on the co‐design and co‐production of the research.^41^, ^42^

### 2.4 Materials

The material collected comprises the transcripts of the meetings conducted during the project and content from the book *Psychiatry as Protagonists*, which was read and signed by all participants before publication. In addition, two interviews were conducted with privileged witnesses before this article was written.^43^, ^44^ The material collected is summarised as follows:

1. Two interviews with the leaders of the two groups, one initial and one final.
2. Analysis of the transcripts of the 30 meetings (15 for Group A and 15 for Group B).
3. Analysis of the conductors' notes from all meetings.
4. An additional 10 interviews with service users.
5. Analysis of the texts of e‐mail correspondence between the participants on the topics discussed in the group.

#### 2.4.1 Data analysis

Content analysis^45^ was used in the process of reading and coding the textual data, which led to the creation of a codebook applied to all responses. Following the analysis, which was carried out independently by the two EbEs and one researcher, any differences were resolved through discussion. A second researcher helped refine the codes and create a common data set. Increasingly detailed categories for analysis were thus identified, starting from the initial macro‐category (corresponding to the topic investigated by the question) to two or more specific categories. After the analysis, all authors of the paper met to discuss the results and make any necessary changes. The draft of the main results summary paper was also shared during the co‐analysis and co‐writing phases.^42^, ^46^

## 3 RESULTS

### 3.1 The initial phase: Critical issues related to accessing mental health services

The first topic that emerged concerned access to mental health services. In both groups, considerable attention was paid to the difficulty of making the first contact. It is important to note that this comment on the different perceptions and attributions of a mental health problem by family members and users was offered by all three roles involved (users, family members, Ebes). The account was made a posteriori: it is the reconstruction of a phase, the one before taking charge, in respect of which a reconciliation of needs followed together with the recognition of illness, witnessed by the taking charge by the service. However, at first, the agreement between family members and users was complicated because the person did not recognise the need for specialist psychiatric counselling, procrastinating and avoiding meetings with people who could have put pressure on them. For many, a lack of awareness of their problem was the foundation of profound initial disorientation and major conflict. The family turned out to be the cause of most misunderstandings and conflicts before initial contact was made. In some cases, the family members were certain that the person in question needed psychiatric assessment, even though that individual did not want to, while in other cases, it was the opposite.

The family relationship is a core element in access and is often the trigger that blows up the situation. For example, when parents do not have the proper tools to make sense of their child's difficulties, they may underestimate them, minimise them or feel ashamed and hide the problem in silence. Sometimes, on the contrary, it is the family that contacts the service when they are unable, for some reason, to have their son or daughter seen by specialists. Conflicts, standoffs and ruptures can arise in such situations. The beginning of the path is often a time of immense loneliness and existential restlessness. The person in distress can experience a profound life crisis, sometimes even leading to their death.

The person who makes the referral to mental health services is often the general practitioner (GP), who may know nothing about mental distress and may not have the proper skills to recognise its symptoms. A conflict could arise between the GP and the person who raised the issue (could be a family member or a distressed person). Often, the GP is not involved in the mental health services network and does not know how the service works in terms of requesting it and is therefore unable to lead the patient confidently towards the service. If the relationship between the GP and the mental health centre were less anonymous and more personalised, and if the doctor knew an operator of the service to whom they could ‘entrust’ the patient, users and their families would likely feel more comfortable and supported. Users are generally frightened of encountering a mental health centre. Because they often feel highly vulnerable and are afraid of losing the power to make important decisions about their own lives, they may oppose the services and be reluctant to admit they have any problems. Thus, to avoid exposing them to a threatening situation, it is vital to humanise the service environment. The following Table 4 shows some of the strategies generated by the two groups as suggestions for how to humanise the service.

**Table 4:** The ideal service: Entry phase.

1. Strategies to facilitate the entry phase	Avoid cold and detached mailings with respect to building a relationship of trust between GPs and the mental health centre. Prepare a leaflet offering information about the mental health centre and make it available to patients in GPs' offices. Have a video presentation of the mental health centre on the service's website. This could present the objectives and actions promoted by the service, and it could also include anonymous voices of former users and relatives to reassure them about their prospective access and experiences. The video could present and enhance the role of the EbE. Ensure the presence of an EbE in the new user's welcoming phase. The user could this be reassured through interaction with ‘another like me’. Offer the family members of the new user support from other family members (in the role of EbEs) to guide them in accessing the service and in the ways to remain close to their loved ones, despite initial difficulties. Reduce waiting times. Expand the availability of EbEs within the service.
2. Strategies to reduce the stigma of mental illness	‘Camouflage’ the mental health centre by eliminating obvious signs that make explicit the terms for mental illness and instead use terminology linked to well‐being (e.g., ‘cure’, ‘prevention’, ‘therapy’, ‘well‐being’, ‘health’ and ‘recovery house’). Guarantee more anonymity for users by providing a secondary entrance to the mental health centre, thus making them less visible and avoiding stigmatisation. Implement projects to counter the stigma of mental illness in schools and services in the area. Organise community events aimed at raising awareness of the service and its functions. Allow users access to the cafeteria within the service centre that is run by ex‐patients. Create service areas for use by the local population to facilitate encounters and help reduce stigma and prejudice, e.g. a café accessible to service users and outsiders.
3. Strategies to improve the physical environment	Provide large and comfortable clinic spaces, with background music, furnished in bright and lively colours. Ensure an appropriate temperature in the rooms and provide good quality furnishings, including plants, photographs (possibly taken by users and operators during a service improvement workshop), and artworks, which all contribute to making the user feel comfortable and at home. Environments typical of hospital facilities, which are anonymous and cold, make patients’ visits more tiring and difficult and hinder their involvement in service activities. Provide adequate equipment (for workshops, manuals or relaxation activities), internal signposting that leads to correct locations and information panels and spaces that can be adjusted according to the users’ needs (with posters, their own objects, etc.). Provide accessible outdoor parking spaces and spaces for family members, such as cafes and a garden for informal meetings, to encourage spontaneous interaction between all service users.
4. Strategies to improve organisational aspects in the reception phase	Avoid inefficiencies related to waiting times, requests that remain blocked or unaddressed, and unavailable emergency telephone services. Fulfil requests as soon as possible and establish a maximum waiting time that cannot be exceeded. Make the referral specialist's e‐mail public, and ensure that mail messages are read and answered efficiently. Give adequate notice in the event of an appointment's postponement or cancellation by service providers. In the clinic, arrange columns with interactive videos on how the service works. These could provide general information about the clinic, instructions on how to move around, staff presentations, relevant activities and favourable outcomes of past recovery stories. Ensure certified organisational procedures that minimise human error. Ensure training courses for operators' improved ability to handle new procedural or behavioural arrangements.

Several observations have been already made about to the vital role of GP, who are the first interlocutors in the user's relationship with the service. However, their role does not stop at the referral; in fact, their collaboration with the service must be continuous and constantly updated in both directions, from and to the service.

### 3.2 The active involvement of the family

3.2.1

One of the thorniest topics in the relationship between families and the service is the confidentiality agreement. An adult service user may request that information relating to his or her medical record not be shared with anyone, including family members. This is especially the case when the user has an ongoing conflict with his or her family. In such cases, the service may confirm, consolidate, or even exacerbate the conflict, as the family remains on the outside, without knowledge of the problems their loved one is experiencing and hence feels excluded from the process. Although in some cases, an initial distance approach may be helpful in maintaining a space of autonomy for the affected individual and in developing critical thinking regarding the user's role, it is necessary that the family be supported and assisted in the process. The care pathway must sooner or later involve the whole family system. If the dynamics remain entangled or dysfunctional, when the user feels a little better, they will return to a ‘problematic and challenging’ relational context, and the improvement will begin to wane. Therefore, the family must be considered an integral part of the therapeutic pathway. However, how can this be ensured? The ideas presented in Table 5 were offered by family members and family EbEs. Moreover, interaction with family members can be a great resource for referring doctors, allowing them to access other points of view, to get to know the user's background and to develop helpful relationships.

3.2.2

**Table 5:** The ideal service: Family involvement.

Strategies for family involvement	Organising meetings for family members on various topics and in different ways. Informing them about confidentiality and what they are about to pursue, increasing their awareness of what the mental health service offers, helping them to recognise the symptoms through the use of various diagnostic pictures and offering them advice on how to sustain their involvement with their loved ones and the service. How to conduct meetings that contain information about the service might vary according to the setting and the aims. The meetings could be led by healthcare personnel, psychiatrists or social workers in collaboration with family members and users. Meetings related to the involvement of the loved one and the service could be guided, or rather advantaged, by EbE family members, who could bring their own stories as examples. This approach could involve an open self‐help group and provide an opportunity for families to break out their isolation, free from the perception of stigma, or simply to share their struggles. A strategy to involve the user, that is individually based rather than group based could enhance the GP as a bridging figure between the service and the family.

Abbreviations: EbE, experts by experience; GP, general practitioner.

### 3.3 Networking with the territory and the university

The participants of the two groups recognised that knowing the territory and its resources is a prerequisite for an effective and efficient recovery path. The recovery experience was defined as a slow but progressive pathway of a patient's reinsertion in the context of relationships on a wide spectrum: family, work, recreation, social and voluntary work. To develop self‐confidence in one's own abilities, individuals need recognition and rewarding roles, not only in private environments but also in institutional ones. For this purpose, one of the ideas that emerged in the groups was the possibility of setting up an office, that is, solely dedicated to networking and liaising with institutions, social services, recruitment agencies and other organisations in the area that could play a major role in the social and employment inclusion of service users. The office should be equipped with the appropriate professional, operational and financial resources.

A second important service component is a research centre from an independent university with the ability to lead the research and provide specific training about the scientific‐disciplinary fields related to the mental health topic (e.g., knowledge of the forms of mental distress, the effects of pharmacological interventions, scientifically tested formulas and protocols for pharmacological deprescription and knowledge about the sociological aspects of the relationship between social inequality and mental distress, etc.). The research centre could also report on the role of EbEs and gather data to test, which could help to assess their specific contributions to the recovery outcomes associated with the services.

In addition, the university could offer the time and resources to develop projects around common interests, to conduct fundraising activities dedicated to new intervention practices, to networking with partners who can create working groups from all over the world, utilising international funding and sharing knowledge based on emerging mental health approaches.

Furthermore, the mental health centre could, in partnership with the university, set up an observatory on the mental health field and its evolution. The presence of an external and independent body, such as a university, could promote systematic studies of the efficacy and efficiency of recovery pathways (e.g., favourable recoveries, discharges, reinsertions with critical or positive outcomes, relapses, suicides, failures and criticality). Universities could also promote this through the systematic organisation of prestigious multidisciplinary conferences that feature professionals who are accomplished in dealing with mental health, medical and social research.

Another topic of great interest, closely connected to the previous ones, concerns the possible offering of training about users to the service operators at various levels, thus providing new knowledge in all medical sectors that are contiguous to the psychiatric one. Individual and group psychotherapy courses could be offered to users, EbEs, and family members that provide them support in times of profound vulnerability. The consultant psychologist could also suggest new ideas to improve the team's working climate and mediate conflicts between families and users. They could encourage the users’ involvement and enhance their cooperation with EbEs.

Other professionals important in the protection of users' well‐being and health are neurologists, nutritionists, sexologists and neuroendocrinologists, all who can provide different information and advice, including psychoeducation courses about the side effects of drugs and how to counter them. Legal advice and regular discussions with social services could also be important resources, regarding knowledge of service users’ rights and their defence and protection.

## 4 DISCUSSION

The first and most important contribution of our research concerns more than ‘what was said’. Rather, it pertains to ‘how what was said was collected’, that is, the management of the project and its ability to counter what the literature defines as ‘resistance’ or ‘barriers’ to the active participation of users or former users. Instead of intervening directly by defining a priori the way in which the initiative would be carried out, the service chose an indirect strategy. It commissioned the task to two EbEs, giving them complete freedom to organise and offering them all the necessary support. The research thus avoided involving psychiatrists or service workers as organisers of the initiative. This approach made independence, autonomy and freedom of expression possible for the users and family members who, far from judgmental gazes of professionals involved in assessing their mental health, felt they could say what they genuinely thought with respect to the critical and fragile points of the system. This made it possible to go well beyond traditional user satisfaction surveys^9^, ^15^ and to overcome the power dynamics that often hinder true and deep collaboration between users and services.^11^, ^12^ The two EbEs who conducted the groups acted as effective mediators between the service and the patients, playing different roles—one was a family member and the other a former service user—further enriching the process and ensuring a broad and comprehensive representation of the different facets of the user's experience.

Furthermore, a crucial element was the independent position of the experts. Although affiliated with the service, they were not directly commissioned by the service but rather operated through an external nonprofit organisation. This ensured a thorough, impartial and frank discussion of the issues, free from possible conflicts of interest and fear of repercussions.^47^ We believe that this article is a successful example of co‐design precisely because it was conducted by individuals affiliated with but external to the service, who could maintain the privacy of the users’ comments.

Regarding the content that emerged in the co‐design, we can distinguish between ideas relating to the implementation of the service generally (ideas that, moreover, had already been made available in the literature, such as those for combating stigma,^7^ for fostering the humanisation and personalisation of care programmes^2^, ^3^, ^4^, ^5^ to protect the users' privacy^48^ and for training^49^, ^50^), and those which instead represent a great novelty for the specific context in which the research was carried out, since they identify activities and competencies of the EbEs in a country where the EbE role in healthcare service delivery does not yet exist (Table 6).

**Table 6:** My ideal service: Summary of EbE‐specific change initiatives.

Strategy for welcoming	The EbEs' role is very important in the welcoming stage. EbEs are an immediate point of contact for the user, not only because they are familiar with the structure of the service system but also because they have previously experienced the challenges and uncertainties related to mental health. This instinctive understanding of the ‘language of the user’ is a powerful benefit, making the service approach less intimidating and more accessible.^11^
Stigma reduction strategy	The EbEs collaborating in the service recognised its enhancement with respect to the users' experiences. Their presence and active involvement not only in training sessions for operators, users and family members, but also in relation to the entire citizenry, offers a strategy of counteracting the prejudices around mental illness. Their stories about the challenges they overcame and their arduous recoveries in the turbulence of their recovery paths can be an effective tool in educating the community about the realities of mental health and in combatting stereotypes that separate people experiencing mental health issues from the rest of society.
Communication and involvement	Another vital aspect to consider is the EbE's role in facilitating communication between users and practitioners.^20^ Through their personal experience, EbEs can act as a bridge between the two parties, assuring that the user's needs are fully understood and considered. This balance in communication is vital, especially regarding decisions about treatment options.
Training and co‐design	The involvement of EbEs in training is a key innovation. With their direct experience and understanding of the difficulties of recovery, EbEs can provide valuable insights for training and awareness‐raising projects in schools. Moreover, their active participation in the training ensures that the approach adopted by the service is truly user‐centred, thus reshaping traditional power dynamics and promoting a collaborative climate.

Abbreviations: EbE, experts by experience; GP, general practitioner.

The lack of recognition of the EbE's role in the Italian context entails many critical issues. As Table 6 shows, EbEs can carry out activities involving many varied skill domains, from listening to supporting to communication (even in public contexts, such as in schools and the public), mediation and organisational skills. Specifically, in phase 1 of our project, it was assumed that the two EbEs could manage two working groups of more than 15 people each for an extended period, and that they had research skills for interviewing users and family members and conducting focus groups. While this ‘natural experiment’—as similarly defined in the pilot study constructed by De Graaff et al.^51^—ensured the involvement of EbEs in the co‐production of services, it should cause us to reflect on the importance of not taking EbEs’ skills for granted. We aimed to present this project as a virtuous but also very demanding and challenging experience, representative of how EbEs are confronted with situations about which they have not received specific and relevant training. They are often called upon to deploy personal and professional skills that are often acquired beyond the official preparation for the role. In addition, the lack of institutional recognition further contributes to the role's fuzziness and uncertainty.^52^, ^53^ On the other hand, as pointed out in the literature^53^ the involvement of EbEs is not in itself a guarantee of collaborative projects. On the contrary, it can be critical to collaborate with different types of knowledge and training if the cornerstones within which the words collaboration and co‐participation can make sense are not shared beforehand.^53^ There must therefore be clarity regarding the objectives and the collaboration between the various roles that democratically contribute to achieving them.

To conclude, it should be noted that, with respect to the collaboration expressed in phase two, that is, in following the approach to collaborative writing in the design and writing of the present article,^54^, ^55^ we have from the outset shared the objectives of the project and worked jointly at each stage, organising times for discussion and interaction between the whole research team. This allowed us to mitigate the barriers to collaboration between academics and EbEs, which were closely related to the dominance of professional epistemology in the production of knowledge about mental health service quality.^51^ Even with respect to this second phase, however, we are aware that the recognition of the role of EbEs and the activation of specific research‐related training proposals, still nonexistent, would ensure more equal relational and collaborative conditions^56^

## 5 CONCLUSION

This article addressed the complex issue of users' first contact with mental health services by proposing a cross‐section of the co‐design of a care pathway regarding how the services can be improved, or rather, ‘what an ideal service should be like’. This study showed that regarding the critical issues highlighted by the users, there is no need for direct intervention, but rather for a process of continuous involvement of individuals who can promote reflexivity in the service.

We emphasised the need for research that will thoroughly analyse both the critical issues related to the inclusion of EbEs in Italian mental health services and the potential improvements to this role that is still under construction and whose definition is still evolving in the Italian context.

This process is not only a fundamental step towards a more inclusive and participatory mental health service, but it also represents a concrete commitment to transform the perceptions and experiences of users within the service, creating an environment that is more conducive to sharing and dialogue. However, this process is not without its challenges. Understanding and integrating the role of EbEs within the existing system requires time, adaptation and training for both EbEs and other professionals involved.

## 6 LIMITATIONS

The first limitation of this study was not having explored in detail how the ‘ideal service’ project affected service improvement. Although the inclusion of group leaders and a member of the service team provided an authoritative perspective on the impact of the project, a more in‐depth assessment of the actual changes resulting from this intervention would have further enriched our conclusions. This article, however, bears witness to the realisation of one of the expressed goals, namely, the much‐desired collaboration between the university and the mental health centre.

Furthermore, our study mainly focused on structuring and implementing the project without fully investigating how the EbE role is perceived in the service context. This is a crucial aspect, especially considering that the role of the EbE has still not been identified and consolidated in the Italian context.

In the future, it will be essential to address these limitations through research that explores both the real impact of initiatives, such as ‘the ideal service’, on mental health services and the perception and integration of EbEs in the Italian health services environment. This will not only allow for a better understanding of the potential and challenges associated with this professional figure but will also optimise any further involvement and co‐design strategies in mental health services.

Among the limitations of the work, the composition of the research team also deserves some note. The study team included three university researchers, two Ebes and a psychiatrist. One might wonder whether the composition of the team met the criteria of equity among the roles required for co‐design.^57^, ^58^

In this regard, we should specify that the EbEs did not have specific research skills, in addition, neither the researchers nor the psychiatrist who commissioned the project were present during the focus groups, the latter never went into the substance of the content or the way the research was conducted. The researchers only collected the material and conducted the interviews at a later stage. Their contribution concerned their competence in analysing the collected material. The number of co‐authors in the various roles involved (1 university researcher and psychotherapist and 2 PhD students, as opposed to the 2 Ebes) is justified by the large amount of material to be codified and the need to join forces for the scientific dissemination of the results.

We must recall that, unlike what happens in other European nations, the figure of the ‘EbE researcher’ does not exist in Italy. Therefore, we believe that our research represents a rare experience that should be valued, precisely because it tells of a first important opportunity for shared research between academia and services on this issue. We understand that, in other countries, the criteria of EbE involvement in the research process are much more refined and equal, but we believe that attention to the specificities of the Italian context, is necessary.

A final limitation is not having distinguished the results according to roles, i.e. between users and family members, preferring to mix them (according to previous research^59^ and to the methodology proposed by FAREASSIEME^38^) to ensure a maximum heterogeneity that could broaden the range of perspectives and ideas. The choice to mix the roles of family member and user may have conditioned the themes expressed and the freedom of speech, however, we believe that the two facilitators of the focus groups, being a family member and a former user, paid great attention to the space of expression of the members of their role category. In future research, built from the beginning in collaboration between EbEs and researchers, we will pay more attention to this issue.^59^, ^60^, ^61^, ^62^

## AUTHOR CONTRIBUTIONS

**Michele Rocelli**: Investigation; methodology; writing—original draft; writing—review and editing. **Ludovica Aquili**: Investigation; methodology; writing—review and editing; writing—original draft. **Paolo Giovanazzi**: Investigation; methodology. **Andrea Puecher**: Investigation; methodology. **Marco Maria Goglio**: Visualisation; supervision. **Elena Faccio**: Conceptualisation; investigation; funding acquisition; writing—original draft; writing—review and editing; methodology; data curation; supervision; project administration.

## CONFLICT OF INTEREST STATEMENT

The authors declare no conflict of interest.

## ETHICS STATEMENT

Approval for the study was obtained from the Ethical Committee of the School of Padova, at the University of Padova (Approval No. 4862). All patients provided written informed consent before enrolment in the study. The participants and their families were informed of their right to withdraw at any time. They signed a written informed consent form regarding their participation in the research and its publication.

## Data Availability

The data that support the findings of this study are available on request from the corresponding author. The data are not publicly available due to privacy or ethical restrictions.
